# Identifiability, Risk, and Information Credibility in Discussions on Moral/Ethical Violation Topics on Chinese Social Networking Sites

**DOI:** 10.3389/fpsyg.2020.535605

**Published:** 2020-10-23

**Authors:** Xi Chen, Chenli Huang, Yi Cheng

**Affiliations:** School of Business Administration and Tourism Management, Yunnan University, Kunming, China

**Keywords:** anonymity perception, risk perception, information credibility, content moderation, real names on social media

## Abstract

One heated argument in recent years concerns whether requiring real name supervision on social media will inhibit users’ participation in discoursing online speech. The current study explores the impact of identification, perceived anonymity, perceived risk, and information credibility on participating in discussions on moral/ethical violation events on social network sites (SNS) in China. In this study, we constructed a model based on the literature and tested it on a sample of 218 frequent SNS users. The results demonstrate the influence of identification and perception of anonymity: although the relationship between the two factors is negative, both are conducive to participation in discussion on moral/ethical violation topics, and information credibility also has a positive impact. The results confirmed the significance of risk perception on comments posted about moral/ethical violation. Our results have reference value for identity management and internet governance. Policies regarding users’ real names on the internet need to take into account the reliability of the identity authentication mechanism, as well as netizens’ perceptions of privacy about their identity and the necessity of guaranteeing content and information reliability online. We also offer some suggestions for future research, with a special emphasis on applicability to different cultures, contexts, and social networking sites.

## Introduction

The complex integration of the internet and the real world means that in both the West and China, cyberspace has become the most convenient place for free expression, which is constrained by social norms and conformity (Lipschultz, 2018). Online public opinion is becoming the mainstream public opinion domain in China (Yu, 2017). China arguably presents an interesting case study on social networking sites (SNS) because it limits social media communication on non-domestic sites, establishing a microcosm of SNS (Sullivan, 2014). The expression of online public opinion is rooted in the social and cultural background of real-life society. In Chinese culture, there has always been an emphasis on “denying self and returning to propriety”, personal behaviors should be “gentle, modest and courteous” and expressions should be humble and low-key (Chen, 2014). In interpersonal communication, a superficial balance of relationship should be pursued, and telling the truth should be avoided to prevent harming interpersonal relationships (Zhai, 1999). In fact, culture is shaped by reality. When real lives are mapped onto virtual cyberspace in a hidden form, this principle of superficial balance is no longer important (Chen, 2018). Since Chinese people lack freedom of expression of their real views with their real-life social contacts, online anonymity is of greater importance to Chinese people compared with those from the West. In an interview survey conducted in 2017, 79.1% of 48 respondents said that they assume different identities online, which is reflected in using different SNS accounts (Chen, 2018).

In the most recent couple of decades, many researchers have regarded anonymity as directly enabling free expression on the internet as well as being the root cause of anomie (Nissenbaum, 1999; Davenport, 2002; Kim et al., 2011; Salanova et al., 2013; Stroud, 2014). The system of using real names online, which is considered a way of enhancing oversight of cyberspace and regulating the behavior of netizens, has been gradually established and improved with the development of China’s internet governance (Lin, 2010; Liu, 2013).

In 2015, the State Internet Information Office of China issued a regulation named Rules on Account Name of Internet Users, which requires all users to submit real-identity registration information when using the internet. With the precondition that internet regulators can confirm users’ identities, users have the right to use virtual names in online public speech spaces, which should be respected (Chen and Li, 2013). The real-name system is a mechanism that enables an individual’s name to be mapped to that person’s identity on social media. Users must provide information on their real personal identity when engaging in online activities, so as to establish a consistent relationship between their online and offline identities, enabling a confirmatory mechanism that links the rights, obligations, and interests of individuals’ words and deeds online and in real life (Chen and Li, 2013).

China’s enforcement of the online real-name registration system sparked widespread and fierce disputes, focused on its impact on netizens’ freedom of expression. Supporters of the regulation argued that the system is conducive to creating a credible online speech environment and encouraging people to be responsible for their own speech. For those who are willing to speak frankly, real-name speech can also improve personal credibility and give weight to their words (Huang and Zhang, 2010). Opponents contend that the real-name system undermines the traditional values of equality, freedom, and openness on the internet, discourages internet users from participation in politics and scrutinizing government, and poses a covert threat to netizens’ right of “freedom of expression” (Zhang and Lu, 2010).

Public concern about things that affect the majority of society is an important force in implementing oversight and promoting social progress. In China, Weibo and WeChat, with 500 million and 1 billion active users respectively at the beginning of 2020, have become the two most important SNS for people to express public opinion, offering different kinds of platforms for open and critical debate (Rauchfleisch and Schäfer, 2015).

In recent years, China has experienced many public opinion incidents online, with some incidents (both online and offline) sparking a great deal of online reaction and widespread discussion. The vast quantity of views freely expressed online by the public on specific topics has promoted social regulation offline, including efforts to promote the optimization of the social system and to combat corruption (Liebman, 2011). This plays an important role in social justice and promoting reform of the system of governance in the real world. The ability to trace a person’s identity magnifies the risk of individual participation in exposing social problems, including interpersonal risks, moral risks, and even security risks, and this is an important reason for interrogating the online real-name system. However, to date studies on identifiability online have failed to explore this aspect. Therefore, the first research question to be tested in the current study is:

RQ1: Does the traceability of network identity information inhibit public participation in discussions on moral/ethical violation topics by internet users in China?

Anonymity in cyberspace is an important way of protecting private information (Brazier et al., 2004; Rainie et al., 2013) and is conducive to the construction of self-image (Lin and Utz, 2017). The emergence of new cyber applications has led to a heated debate over the advantages and disadvantages of anonymity in cyberspace (Chen et al., 2016, 2019a, b; Christopherson, 2007; Lapidot-Lefler and Barak, 2012; Scott and Orlikowski, 2014; Fox et al., 2015; Jardine, 2015; Levontin and Yom-Tov, 2017). A series of research studies have confirmed that the perception of anonymity has different impacts on behavior in different online environments (Jessup et al., 1990; Joinson, 2001; Reinig and Mejias, 2004; Lapidot-Lefler and Barak, 2012; Yoon and Rolland, 2012; Hsieh and Luarn, 2014).

In the current cyberspace environment, absolute anonymity does not exist (Bodle, 2013). The issue of anonymity is often the focus of research on free expression (Akdeniz, 2002). SNS, especially those in which users tend to use real names, such as Facebook and WeChat, provide users with the freedom to make choices; the social connections built and maintained by these platforms may reduce the perception of anonymity. The positive impact derived from a perception of anonymity on positive self-disclosure has been analyzed in detail (Chen et al., 2016). Positive self-disclosure relates to the construction of self-image. However, participation in the discussion of topics that violate ethics is related to social responsibilities. Everyone has the responsibility to assume the ethical responsibilities of the media (Boeyink and Borden, 2010). For Chinese who value harmony of interpersonal relationship and the dignity, online anonymity has become a “veil”, creating conditions whereby they can express their opinions freely. Anonymity in cyberspace is of great significance for Chinese netizens to express free speech about their true views. There is a lack of analysis in the current literature on the impact of perception of anonymity on users’ participation in assuming public social responsibilities. Therefore, the current study attempts to answer the following research question based on the SNS environment in China:

RQ2: Does the perception of anonymity on the internet help drive netizens’ public participation in discussions on moral/ethical violation topics in China?

Based on the understanding of the issues described above, we synthesized the existing literature to build a theoretical model, as well as referring to the theories of the social identity model of deindividuation effects (SIDE) and Borden’s communication ethical rules. Empirical research was conducted to test the hypothesis and theoretical model. The current study makes two principal contributions to the literature. First, it reveals that identification and perception of anonymity are opposite aspects of the influence mechanism of online participation in discussions on moral/ethical violation topics, and encouraging such participation needs to take into account underlying aspects of identification and the psychological perception of anonymity at the surface. Second, it confirms the role of risk perception and information credibility in participation in discussions on moral/ethical violation topics.

The remainder of the paper consists of the following sections: Section 2 reviews the theoretical foundation, and Section 3 proposes our research model and hypotheses. Section 4 explains the methodology, while Section 5 presents the results and related analysis. Section 6 discusses the results of the current study, together with the theoretical and practical limitations and potential avenues and implications for future research.

## Theoretical Foundation and Literature Review

### Cyber Anonymity and Online Public Opinion

Anonymity means a lack of identification of one’s real identity (Marx, 1999). As a result of the integration between the internet and the real world, identification of users’ real identity has become the basis for internet services and governance in China (Chen, 2018). According to the social identity model of deindividuation effects (SIDE), in the context of anonymous identities, people show a behavioral tendency to obey a group norm due to the prominence of an individual identity (Vilanova et al., 2017). The effects of online anonymity shown in the SIDE model are reflected in personalization, misconduct, and false information, which are related to the dark side of cyberspace (Fox and Moreland, 2015). However, anonymity in cyber-based communication may not necessarily lead to antisocial behavior (Christopherson, 2007). In some scenarios, anonymity enhances social processes related to group identity in online communication (Spears, 2017). On SNS, anonymity can also play a positive role in information exchange (Yoon and Rolland, 2012; Chen et al., 2016).

In the 1990s, use of cyber-based communication technology facilitated an anonymous communication environment, but this positive outcome is no longer the case (Chen et al., 2016). This is because anonymity is now seen by some as dangerous due to the following factors: issues in the protection of business transaction security (Chen et al., 2019a); government oversight and control; concerns about intellectual property; national and international legal implications; and the use of identity management technology (Froomkin, 2015). In SNS, user identification takes complicated forms, with complex and diversified functions and methods of interpersonal interactions.

The importance of online opinion, also called online word of mouth (e-WOM), has been confirmed (Goldsmith and Horowitz, 2006; Weeks et al., 2017). Despite the increasing disappearance of anonymity on the internet, it is still an important “safety valve” for the oppressed, dissidents, and whistleblowers to speak freely (Froomkin, 2015). This ability often comes from the psychological perception that they can engage in free speech without fear of the consequences (Chen et al., 2016). Therefore, online opinion makers can continue to participate, innovate, and explore topics and issues with a high degree of self-cognition. They boast stronger computer skills and use the internet more frequently (Lyons and Henderson, 2005).

Many studies have demonstrated a positive relationship between internet use, online speech, and political participation (Shah et al., 2005; Van den Eijnden et al., 2008; Valenzuela, 2013; Boulianne, 2015). In China, although government censorship inhibits people’s willingness to voice their opinions to some extent, thanks to its loose network structure, which provide users with flexible expression forms and places to disclose opinions, the internet has still led to progressive changes in Chinese society (Shen et al., 2009). Public opinions on the internet affect the real world through users’ discussion of specific events and dissemination of information (Yue et al., 2017). The main participants in cyberspace include stakeholders and the public (Zhang et al., 2015). The methods of participation include providing information, making comments, and involvement in decision-making or particular behaviors. The results of participation affect public decision-making or governance behaviors. Researchers have studied the impact of cyber anonymity on self-disclosure and information sharing (Yoon and Rolland, 2012; Chen et al., 2016, b).

China is in a social transformation period, bringing a high degree of uncertainty to people’s lives. The prevalent practice of concealing their true views for self-protection makes Chinese netizens present more complex mentalities and more diversified modes of behavior than before the construction of cyber identity (Chen, 2018). This also suggests that anonymous expression online plays a crucial role in alleviating potential pressure in real society and relieving the latent contradictions and conflicts. However, no empirical research has been conducted on the impact of a lack of anonymity on expression of public opinion on moral/ethical matters. The exploration of this impact mechanism is an important basis for establishing identity management and carrying out governance in cyberspace.

### Participation in Moral/Ethical Oversight

The development of global media brings urgency to intercultural communications on ethics-related topics (Borden, 2016). Cyberspace transfers the function of traditional media and its societal influence from professional journalists to every netizen. Every speaker in cyberspace has the function of the media to some extent. Therefore, the discussion of ethical and moral responsibility in journalism theory provides an important reference point for individuals’ posting information and sharing behavior on SNS. From a moral/ethical standpoint, journalists have to be clearly aware of what they are and what they are not, and whether they are to stand in favor of some things and against others (Borden, 2007). Media ethicist Elliott (1986) suggests that three levels of responsibilities provide the foundations for moral excellence in journalism: general responsibilities, particular responsibilities, and individuals’ personal responsibilities.

Media participants should follow three ethical rules: truth telling, privacy, and fairness (Boeyink and Borden, 2010). In some controversial ethical violations, the three principles may come into conflict (Boeyink and Borden, 2010). However, in some cases involving ethical and moral principles in which people reach a consensus, the behavior of paying attention to and getting involved in the discussion itself is in compliance with the above three principles. Moral excellence consists of performing your ethical responsibilities well: all of us have moral responsibilities, such as to be truthful to avoid harming others and to keep our promises, so called general responsibilities, which matter in everyone’s lives. These responsibilities should give individuals the power to supervise and condemn those behaviors that violate morality and ethics and endanger the foundation of human existence.

In cyberspace, netizens participate in discussion of an event to supervise and condemn behavior which violates norms of ethics and morality (Repnikova, 2017). The power of moral supervision plays an important role in aspects such as maintaining social justice, promoting improvements in the social system, and restraining corruption (Liebman, 2011). Even though our individual actions are constrained by general and particular responsibilities, media participants have to retain autonomy as moral agents (Elliott, 1986). In particular, when events occur that violate the universal morality of humanity, the involvement of people in discussion and information sharing in cyberspace strengthens the argument for justice; thus, such participation plays an important role in maintaining universal ethics and the morality of the social system. The current study also discusses how the concealment and revelation of individuals’ real identity in cyberspace affect users’ involvement in moral supervision.

### Perceived Risk and Information Credibility Sources

Studies of risk perception examine the judgments that people make when they are asked to characterize and evaluate hazardous activities and technologies (Slovic, 1987). Perceived risk has been conceptualized in terms of the expected negative utility of particular actions (Peter and Ryan, 1976). The impact of perceived risk on behavior has been confirmed in online research contexts, including information sharing and control (Gerlach et al., 2015; Hajli and Lin, 2016) and adoption behaviors (Featherman and Pavlou, 2003; Horst et al., 2007; Martins et al., 2014; van Winsen et al., 2016). Perceived risk reduces users’ perceptions of value (Snoj et al., 2004; Chang and Tseng, 2013) and destroys trust (Slovic, 1993). However, the influence of perceived risk on the expression of ethical views has been neglected in current research.

Perceived risk has an impact on people’s moral judgments Subjects in a high-risk treatment group exhibited significantly harsher ethical judgments than those in a low-risk treatment group (Cherry and Fraedrich, 2002). Reputation, a sense of belonging, and satisfaction from helping others are significantly related to e-WOM intention (Cheung and Lee, 2012). Paying attention to moral views and participating in social media posts and sharing this kind of information is of concern for the collective interest (Earle and Siegrist, 2006). The discussion of moral issues online is related to the altruistic punishment mechanism of human behavior, which holds that individuals voluntarily take risks and pay costs for punishing people who violate social norms, and this plays an important role in the evolution of social cooperation (Fehr and Fischbacher, 2004; Boyd et al., 2010).

The framing of risk depends on the media used to perceive it (Ericson and Doyle, 2003). In seeking information, people rely on information sources to build trust, which is in play whenever users exchange information, and the information source—the trusted party—may have a moral responsibility to an information seeker (Hertzum et al., 2002). Information credibility has become an important topic as the internet has become increasingly ubiquitous (Kelton et al., 2008). The influence of trust in digital information has been confirmed as a key mediating variable between information quality and information use (Pan and Chiou, 2011), but the influence of information credibility on information related to discussions on moral/ethical violation topics needs further clarification.

## Research Model and Hypothesis

**FIGURE 1:**
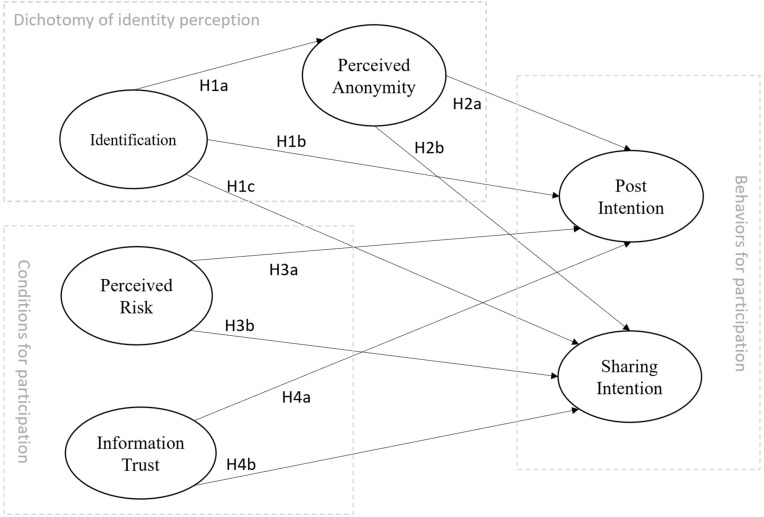
Research model.

The current study constructs a model to examine how the factors of identity perception, real names, and perceived anonymity affect the intention to participate in SNS discussions on moral/ethical issues in China. In addition, the current study also explores the influence of risk perception and information credibility on participation in social media discussions on moral issues in China. The research model is depicted in Figure 1.

### Identification and Anonymity on Social Networking Sites

User identifiability and perception of anonymity are not two sides of the same factor. Although they have opposing properties, they are two different factors. The ability to identify internet users refers to the process and the potential for identifying the true identity of users in cyberspace; this is not just a legal concept but also a technical means of identity detection with the help of publicly available methods (Krausová, 2009). In cyberspace, user identifiability is represented by the richness of information in terms of whether there are clues to determine a user’s real identity (Chen et al., 2019a). Identifiability is objective, whereas perceptual anonymity is subjective and is the psychological perception of the nature of the subject. Previous research has confirmed that identity has a significantly negative impact on perceptual anonymity on various online social media platforms (Chen et al., 2016, 2019a, b). The relationship between these two factors should be the same in monitoring and participating in discussions on moral/ethical violation topics on online social media. Thus, we hypothesize that:

H1a: User identification has a negative impact on their perception of anonymity in discussing moral/ethical violation topics on Chinese SNS.

Identity is perceived as a social process that aligns with internal self-identification and external identity classification (Jenkins, 2014). Identification and self-efficacy are closely intertwined, and the connection between social identity and self-efficacy is further supported by social identity theory (Guan and So, 2016). Four factors—performance accomplishments, vicarious experience, verbal persuasion, and emotional arousal—contribute to a boost in self-efficacy (Bandura, 1977). Social influence and perceived control will positively impact self-disclosure in SNS (Cheung et al., 2015). External factors, such as environment and information input, appear to affect self-efficacy through their influence on internal variables, such as motivation, ability, or performance strategies (Gist and Mitchell, 1992). The identity construction of the user depends on the creation and sharing of information. Attention to and discussion of online public opinion are also a way for the user to construct his or her own identity. The more active the user is on online social media, the deeper the recognition of the user’s online identity and thus the greater enthusiasm and self-efficacy the user has for participating in discussion on the topic. Thus, we hypothesize that:

H1b: User identification has a positive impact on comment intention in discussing moral/ethical violation topics on Chinese SNS.

H1c: User identification has a positive impact on sharing intention in discussing moral/ethical violation topics on Chinese SNS.

Perception of anonymity refers to the indiscernibility of the identity of the user, which leads to self-awareness of identity anonymity, that is, that one cannot be tracked in cyberspace (Kang et al., 2013). In an anonymous environment, social bonds are weaker, and social norms tend to be enforced more aggressively (Wright, 2014). The relationship between perception of online anonymity and behavior depends on the specific communication context (Joinson, 2007). In group discussions, it has been found that users who perceived anonymity were more likely than identified users to embellish the opinions of others (Jessup et al., 1990). For users, circumventing the possibility of authentication can protect privacy (Brennan et al., 2012). Although online anonymity introduces uncertainty into interpersonal interactions, it also reduces risks in online privacy and security (Rainie et al., 2013). According to the SIDE theory, within an anonymous context, people tend to comply with collective norms. Following the argument that general moral/ethical principles lead to collective behavior and consensus (Boeyink and Borden, 2010), the SIDE effect will promote the individual’s obedience to collective behaviors. In discussion and online decision-making on certain sensitive topics, online anonymity increases behavioral contributions and effective suggestions (Jessup et al., 1990). In addition, research on perception of anonymity in the use of online social media for information sharing found that perception of anonymity has a positive effect on self-disclosure (Chen et al., 2016). Thus, we hypothesize that:

H2a: Users’ perceived anonymity has a positive impact on comment intention in discussing moral/ethical violation topics on Chinese SNS.

H2b: Users’ perceived anonymity has a positive impact on sharing intention in discussing moral/ethical violation topics on Chinese SNS.

### Perceived Risks Online

Perceived risk has been conceptualized in terms of the expected negative utility of actions (Peter and Ryan, 1976). Risk discourse is redolent with the ideologies of mortality, danger, and divine retribution (Lupton, 1993). Participation in familiar activities has a tendency to minimize the probability of bad outcomes (Douglas, 2013). Decisions about risk as moral decisions are made in the context of uncertainty (Adams, 2003). However, risk perception has different influence mechanisms in play in discussions on moral/ethical violation topics. The increase in risk entails an attendant enhancement of new moral responsibilities at multiple levels in a society (Ericson and Doyle, 2003). From a moral/ethical standpoint, the media participant has to be clearly aware of their responsibilities (Borden, 2007); in general moral/ethical events in particular, especially the event challenging the basic value and living of human being. the basic principles should be clear (Elliott, 1986). Three ethical rules—truth telling, privacy, and fairness—may come into conflict (Boeyink and Borden, 2010), and should be taken in consideration. Even though our individual actions are constrained by general and particular responsibilities, media participants have to retain autonomy as moral agents (Elliott, 1986). We believe that perception of risk in cyberspace should have a positive impact on participation in discussions on moral/ethical violation topics. Thus, we hypothesize that:

H3a: Users’ perceived risk has a positive impact on comment intention in discussing moral/ethical violation topics on Chinese SNS.

H3b: Users’ perceived risk has a positive impact on sharing intention in discussing moral/ethical violation topics on Chinese SNS.

### Information Credibility

Trust in technology is constructed in the same way as trust in people (McKnight, 2005). Information credibility is a descriptive factor of perceived information quality which influences information exchange. Information quality during an exchange can help build trust and reduce perceived exchange risk (Nicolaou and McKnight, 2006). External factors, such as environment and information input, appear to affect self-efficacy through their influence on internal variables (Gist and Mitchell, 1992). Self-efficacy factors, such as perceived performance, have been confirmed as having an adverse impact on the adoption of e-services (Featherman and Pavlou, 2003), and are also positively related to a consumer’s trust expectation (Hong, 2015). The perceived trustworthiness of information determines the level of confidence developed by the user and the corresponding willingness to use the information (Kelton et al., 2008). Research has also determined the importance of trust in forecasted information sharing in television, newspapers, and online news (Kiousis, 2001), and supply chains (Özer et al., 2011). The source of information is an important factor in considering information credibility (Lucassen and Schraagen, 2010). Morality−relevant information provides the check for value similarity and generates trust (Earle and Siegrist, 2006). The impact of trust on forecasted information sharing has been confirmed (Zimmer et al., 2010; Ha and Ahn, 2011; Özer et al., 2011). Thus, we hypothesize that:

H4a: Users’ information credibility has a positive impact on their comment intention in discussing moral/ethical violation topics on Chinese SNS.

H4b: Users’ information credibility has a positive impact on sharing intention in discussing moral/ethical violation topics on Chinese SNS.

## Methodology

The internet is an ideal medium for collecting data from different groups (Koch and Emrey, 2001). The current study focuses on Chinese SNS users’ participation in discussions on moral/ethical topics. In China, WeChat and Weibo are the most popular online social platforms that people use to express their opinions (Hou et al., 2018). According to the Social Global Web Index’s flagship 2018 report on the latest trends in social media, Facebook is the world’s largest SNS with more than 2.6 billion users, WeChat is ranked fourteenth, and Weibo is ranked eighteenth; the latter are also the only two Chinese social media platforms listed in the global top 20 (Global Web Index, 2018). At the end of 2019, WeChat had over 1 billion active users around the world. Weibo, which has more than 500 million active users, provides a virtual public space for users to share their opinions with their connected peers, making it the most influential opinion platform in China; this makes it a suitable arena for our research on participation in discussions on moral/ethical topics. To validate our hypotheses, we conducted a survey on the use of WeChat and Weibo by Chinese users.

The current research is based on a heated event which provoked discussion all over the world, in which a scientist named He Jiankui announced his work on editing the genes of a fetus. Scientists and authoritative academic institutions from different countries gave their opinions, arguing that He Jiankui had seriously violated academic morals and the code of conduct. What he did also caused an outcry in the international community (Cyranoski and Ledford, 2018; Normile, 2018). His behavior did not only violate scientific ethics, but also had the possibility of polluting the human gene pool and posed a threat to the future of humanity. It was noted that the ethical infractions in this work are among the most egregious that have been recorded in modern medical history since the Second World War (Kuersten and Wexler, 2019). This ethical and moral violation event is significant for the whole of humanity.

Data were collected for the current study at the point in time when this gene-editing of a fetus had just occurred, which had attracted the attention of the whole world and had become a heated topic on various SNS. This was important for focusing the participants’ attention on the research issues, and to obtain a clearer understanding on the event. The administered questionnaire consists of three parts, the first of which is a privacy and protection statement and informed consent declaration. The participants read the information carefully and confirmed it. The second part consists of two news reports about the gene-editing fetus event from *People’s Daily*, which is the most authoritative official media source in China, and the *Beijing News*, which has a wide influence and is based in Beijing. The reports (a total of 884 words) described the development of the event up to December 18, 2018, providing the objective facts calmly and without emotional appeals. The third part required the participants to complete a survey about the gene-editing situation and their feelings about it.

### Data Collection

At the preliminary stage, the current study tested the research model through an investigation of frequent SNS users in China. The aim was to answer the research questions about how users’ identity impacts participation in online speech on moral/ethical violation topics on SNS. According to the relevant institutional and national guidelines and regulations, ethics approval was not required. First, the data collection of the current study did not involve implication, drugs, or mental manipulation, as the participants were only required to report their experiences and behavioral tendency according to their SNS use conditions. Thus, no issues with respect to safety, health, or protection of rights and interests were involved. Second, the data collection required no identity concealment, as no privacy or sensitive issues were involved. Third, the questionnaire for collecting the data in the current study includes an informed consent statement, and the participants were only requested to answer questions according to their SNS use conditions. The data were only to be used for scientific research, without influencing the privacy, reputation, living conditions, or health of the participants. Fourth, the potential participants were offered anonymity; they were fully aware of this option before, during, as well as after giving their responses. Fifth, the current study did not store or use the private information of participants, and any information that may lead to identity risks (only the IP address) was removed during the analysis and submission for scientific review.

The data were collected with a questionnaire using a sample service provided by an online survey platform (wjx.cn/sample/service.aspx). This is the largest online survey agency in China, providing 2.6 million sample banks consistent with the demographic distribution of China’s netizens. The survey employed a purposive sampling method focused on the frequent users of Chinese SNS. Based on user requirements, the platform sends the invitation email to randomly selected potential participants from the sample banks. The survey would begin once the potential participants clicked the URL in the email. First, they were asked to read a news article about the topic, and then they were asked to fill in a questionnaire online. To identify frequent users of social networking platforms for the study and to confirm the quality of data, we included screening questions and limited the response time as a filter, which yielded a total of 218 valid questionnaires out of 345 responses. The service provider was paid 1 USD for each valid sample. We used the SmartPLS to conduct empirical research. One of advantage of using SmartPLS is the sample size, Some SEM based methods need samples of at least 200 samples or more, but SmartPLS is suitable for sample sizes of less than 200 (Sander and Phoey, 2014). Table 1 shows the demographic characteristics of the respondents to the survey.

**TABLE 1:** Demographic characteristics of respondents.

Characteristic		Number (%)
Gender	Female	149 (68.3)
	Male	69 (31.7)
Age (y)	Younger	0 (0)
	15–24	56 (25.7)
	25–34	109 (50.0)
	35–44	38 (17.4)
	45–54	12 (5.5)
	Older	3 (1.4)
User history	< 1 year	0 (0)
	1–3 years	3 (1.4)
	3–6 years	45 (20.6)
	6–10 years	90 (41.3)
	> 10 years	80(36.7)
Education level	Primary school and below	0 (0)
	Junior middle school	1 (0.5)
	Senior middle School	5 (2.3)
	Technical Secondary school	6 (2.8)
	Junior college	37 (16.9)
	Bachelor’s degree	147 (67.4)
	Master’s degree	21 (9.6)
	Ph.D.	1 (0.5)
	Other	0 (0)
Marital status	Unmarried	92 (42.2)
	Married	125 (57.3)
	Divorced	1 (0.5)

The sample had a reasonable demographic distribution. Referring to the data in reports published by affiliated companies Sina and TenCent on Weibo and WeChat in 2018, the age distribution characteristics of the sample were basically consistent with those of Weibo and WeChat users. In the sample, respondents with a bachelor’s degree or above accounted for 77.5% of the total; the education level was slightly higher but within a similar range to that of Sina Weibo users in the reports, 70.8% of whom had university degrees. In WeChat (64% male users) and Weibo (57% male users), the proportion of male users was higher than that of female users. Although the gender distribution of samples may generally lead to bias in the results, gender differences do not affect research on general online sharing behaviors, as confirmed by some related studies on gender distribution differences (Yoon and Rolland, 2012; Cheung et al., 2015; Yan et al., 2016). In addition, data from the current study show that SNS users prefer to browse information rather than publish information. The participants spend on average 81.2% of their time browsing information and 18.8% of their time publishing information and participating in discussions.

### Measures

All measures were adapted from well-established scales, the validity of which had been confirmed in the relevant existing literature. Multi-item measures were applied to ensure the validity and reliability of the study. To ensure comprehension by Chinese users, we translated the scale into Chinese and then back-translated it into English. We asked two researchers to verify the consistency of the terms used in the scale to ensure that the translation and terms were consistent. The scale was modified slightly to fit the SNS context. A seven-point Likert scale (1 = strongly disagree to 7 = strongly agree) is used in all measures. Table 2 lists the constructs and measures applied in the research, as well as the source references. The psychometric properties in Table 2 include Cronbach’s α, composite reliability (CR), and average variance extracted (AVE) of the constructs, as well as the loading, *T*-value, mean, and standard deviation (SD) of the measure items used in the current study.

**TABLE 2:** The measures and psychometric properties.

Items	Loading	*T*-value	Mean	SD
**Identification (Chen et al., 2019a) (Cronbach α = 0.828; CR = 0.886; AVE = 0.661)**				
ID1: I revealed my real name on my social media account. (dropped)				
ID2: I may reveal my name in the messages I post on the social media account.	0.761	20.125	4.252	1.616
ID3: You’ll probably know who I am from my social media accounts.	0.858	43.521	4.344	1.602
ID4: The content I post on my social media accounts is very personal, and it’s easy to tell who I am.	0.867	49.199	3.940	1.548
ID5: I revealed some social information about myself in my social network account, such as company, age, occupation, and hobbies.	0.761	22.251	4.477	1.609
**Perceived Risk (Chen et al., 2016) (Cronbach α = 0.873; CR = 0.897; AVE = 0.638)**				
PR1: I am concerned that participating in this discussion will adversely affect my personal fortunes.	0.738	5.243	3.784	1.386
PR2: I am concerned that participating in this discussion will adversely affect my use of this account.	0.717	4.259	4.032	1.516
PR3: I am concerned that participating in this discussion will adversely affect my personal safety.	0.815	5.982	3.642	1.779
PR4: I am concerned that participating in this discussion will adversely affect my mental state.	0.847	7.708	3.408	1.646
PR5: I am concerned that participating in this discussion will lead to backlash from my family, friends, and acquaintances.	0.864	7.092	3.500	1.654
**Information Credibility (Li and Suh, 2015) (Cronbach α = 0.875; CR = 0.914; AVE = 0.726)**				
IC1: I think the source of this incident is believable.	0.837	29.293	4.752	1.178
IC2: I think the source of this information is usually factual.	0.885	51.474	4.789	1.296
IC3: I think the source of the information about this incident came from credible sources.	0.819	15.792	4.601	1.365
IC4: I think the source of this information is trustworthy.	0.867	44.161	4.638	1.359
**Perceived Anonymity (Hite et al., 2014) (Cronbach α = 0.883; CR = 0.919; AVE = 0.739)**				
PA1: I believe that people who can see what I post or share don’t know who I am. (dropped)				
PA2: I think that people who can see what I post or share don’t know who I am.	0.839	30.401	3.688	1.466
PA3: It is likely that my account will reveal who I am. *	0.870	53.487	3.417	1.510
PA4: Some one else who could see my posting would know my true name. *	0.880	49.815	3.422	1.587
PA5: My personal identity can be guessed by others. *	0.850	34.114	3.252	1.531
**Comment Intention (Jang et al., 2016; Kwon, 2020) (Cronbach α = 0.784; CR = 0.860; AVE = 0.606)**				
bCI1: I will try to post comments on this event.	0.768	23.405	4.193	1.408
CI2: I tend to comment on my friends’ post.	0.828	34.585	4.193	1.440
CI3: I intend to comment on the event more frequently.	0.763	18.592	4.587	1.428
CI4: I will always make an effort to comment on it.	0.754	16.273	3.954	1.667
**Sharing Intention (Chung et al., 2016) (Cronbach α = 0.892; CR = 0.925; AVE = 0.756)**				
SI1: I am inclined to forward reports on the incident to others on my SNS.	0.860	35.704	4.413	1.525
SI2: I tend to post this event to let others on my SNS know about it.	0.845	35.737	4.560	1.517
SI3: I will share this event to let others on my SNS know about it.	0.906	73.530	4.101	1.686
SI4: I usually spread news about this event to others on my SNS.	0.865	42.843	3.982	1.684

*Reverse scale.

## Results

We used a structural equation model to verify the research model and performed statistical analysis using the partial least squares method (PLS). In addition, we used Smart PLS version 3 (Ringle et al., 2014) to test the research model empirically; this is an analytical technique widely used in social science research because it provides a flexible and exploratory method with coherent explanations of complex relationships (Henseler et al., 2014). In accordance with the two-step analysis method (Hair et al., 2006), we tested the credibility and validity of the measured values and then evaluated the structural model.

In the next sections, we analyzed the data in two steps: first, the measurement and data were tested for reliability and validity, and then we drew conclusions about the structural relationship based on the measurement instruments with desirable psychometric properties.

### Reliability and Validity of the Measurement Items

As shown in Table 2, all the indicator loadings were significant and higher than 0.70, except ID1 and PA1, whose loadings were lower than 0.7; therefore, we dropped them, ensuring the convergent validity of the measurement model. The resulting Cronbach’s α of each construct exceeds the recommended level (0.70), and the composite reliability is higher than 0.80, indicating that the reliability of all latent variables is very good. In addition, each variable has good polymerization validity, because the AVE of all latent variables surpasses 0.6.

Ensuring discriminant validity requires a low correlation between measures and other structural measures (Fornell and Larcker, 1981). In Table 3, the main diagonal value is the square root of AVE and the out-of-diagonal value is the correlation coefficient between the constructs. All the diagonal values are higher than 0.7 and exceed the correlation between any pair of measures. This value indicates that the model also has good discriminant validity. Therefore, the results of our data analysis have adequately high discriminant validity.

**TABLE 3:** Correlation matrix and psychometric properties of key constructs.

	ID	PR	IC	PA	CI	SI
Identification (ID)	(0.813)					
Perceived Risk (PR)	0.064	(0.799)				
Information Credibility (IC)	0.263	−0.069	(0.852)			
Perceived Anonymity (PA)	−0.587	−0.023	−0.069	(0.860)		
Comment Intention (CI)	0.242	0.205	0.365	0.053	(0.779)	
Sharing Intention (SI)	0.301	0.137	0.337	−0.022	0.622	(0.869)

*SQRT (AVE) is in parentheses. Off-diagonal cells show the correlations between constructs.*

### Structural Model

**FIGURE 2:**
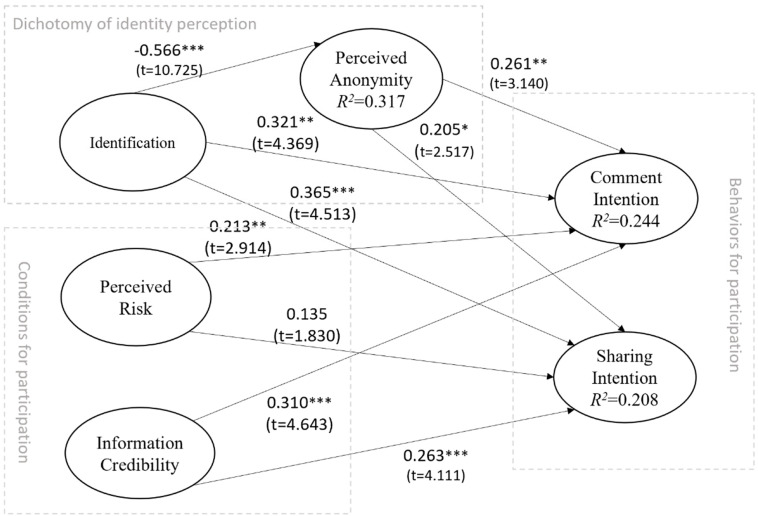
Results of the research model.

Before testing the hypotheses, multicollinearity regarding the structure of the data was tested and was in accordance with the requirements. We then examined the structural model by analyzing the significance of the path coefficients and the R2 variance for the dependent constructs based on the hypothetical research model. The path and its importance for the structural model, the coefficients of each related structure, as well as their *T*-values on the structural model and the deterministic coefficients (R2) are illustrated in Figure 2.

For the full model, most proposed hypotheses are strongly supported by empirical evidence with significance at *p* < 0.05, except for H3b. In this section, we discuss how each construct in our theoretical framework influences the two types of participatory behaviors. Regarding identifiability, we found that identification has a strongly negative influence on perceived anonymity (β = −0.566, *p* < 0.001). This finding is consistent with results in the previous literature; those people who are objectively identifiable will not perceive themselves to be anonymous. Thus, H1a is supported. The results also show that identification leads to participatory behaviors in discussion on moral/ethic violation topics, both in terms of comment intention (β = 0.321, *p* < 0.01) and sharing intention (β = 0.365, *p* < 0.001). In the SNS context, the more personal the information is that is disclosed, the more likely the user is to attend to the discussion and carry out moral/ethical supervision. Therefore, H1b and H1c are supported. Perceived anonymity also leads to participation in discussion on related topics, both in terms of comment intention (β = 0.261, *p* < 0.01) and sharing intention (β = 0.205, *p* < 0.05). Perceptions of anonymity also declaim importance in participation in related activities. Therefore, H2a and H2b are supported. Furthermore, the coefficient of the path from identification to sharing intention is greater than that from identification to comment intention. However, the coefficient of the path from perceived anonymity to comment intention is greater than that from identification to sharing intention. Sharing related information is helpful for the user’s image, while making comments carries less interpersonal pressure. This finding suggests that identification and perceived anonymity both have a positive impact on participation in discussion on moral/ethic violation topics, but through a different influence mechanism.

Regarding the conditions for participation, perceived risk has a significant impact on comment intention (β = 0.213, *p* < 0.01), which is in line with the altruistic punishment mechanism of human behavior. Thus, H3a is supported. However, perceived risk has an impact on sharing intention with a significance level of *p* = 0.067, suggesting that H3b is not supported in the current study, while the direction of the impact is consistent with H3b. We can say that at the test standard of *p* < 0.05, the empirical research cannot significantly support H3b. Regarding perceived risk, people are more willing to comment on ethical and moral violations than to share information. From actual experience, the degree of exposure to information sharing in social networks is higher than that of commenting on information released by others. Without any doubt, information credibility leads to comment intention (β = 0.310, *p* < 0.001) as well as sharing intention (β = 0.263, *p* < 0.001). Trust in source information leads to confidence in a user’s participation in discussion on related topics. The results imply the importance of information credibility even in moral/ethical violation topics. Therefore, H4a and H4b are supported.

The independent variables explain a substantial portion of the variance in the dependent variables. In the current model, identification explains 31.7% (*r*^2^ = 0.317) of the variance in perceived anonymity, 24.4% (*r*^2^ = 0.244) of the variance in comment intention with a significant impact from identification, perceived risk, information credibility, and perceived anonymity, and 20.8% (*r*^2^ = 0.208) of the variance in sharing intention with a significant impact from identification, information credibility, and perceived anonymity.

In Smart-plus, Standardized Root Mean Square Residual (SRMR) and the Normed Fit Index (NFI) may assess the model fit. For SRMR, the recommended value should be lower than 0.08; NFI values between 0 and 1 are recommended. For the current model, SRMR is 0.067 and NFI is 0.781. The goodness of fit value of the model is 0.577, which is significantly higher than the standard of substantial fitting, in which 0.36, 0.25, or 0.1 can be described as, respectively, substantial, moderate, and weak (Marsh et al., 2005). The indices indicate an acceptable model fit of the data.

The results of direct effect (DE), total effect of each construct, and the results of indirect effects existing in the model, as well as the standard error and *T*-values of each effect are given in Table 4. The results show that all direct effects, except for the non-significant direct effect of perceived risk on sharing intention and the significant negative effect of identification on perceived anonymity (DE = −0.566), are positive and significant to varying degrees. Identification has the largest direct impact on comment intentions (DE = 0.321), followed by information credibility (DE = 0.310) and perceived anonymity (DE = 0.261), and perceived risk has the least direct impact on comment intention (DE = 0.213), but is still significant at *p* < 0.01. Among the direct influences on sharing intention, identification has the largest influence (DE = 0.365), followed by information credibility (DE = 0.263), and perceived anonymity has the smallest influence (DE = 0.205), but is still significant at *p* < 0.05.

**TABLE 4:** Direct, indirect and total effect (Bootstrap = 2000).

Effect Types		Effect Mean	S.E.	*T*-value	*P*-value
**Total Effect**	ID→PA	−0.566	0.053	10.725	0.000
	ID→CI	0.173	0.075	2.299	0.022
	ID→SI	0.249	0.074	3.364	0.001
**Direct Effect**	ID→PA	−0.566	0.053	10.725	0.000
	ID→CI	0.321	0.073	4.369	0.000
	ID→SI	0.365	0.081	4.513	0.000
	PR→CI	0.213	0.073	2.914	0.004
	PR→SI	0.135	0.074	1.830	0.067
	IC→CI	0.310	0.067	4.643	0.000
	IC→SI	0.263	0.064	4.111	0.000
	PA→CI	0.261	0.083	3.140	0.002
	PA→SI	0.205	0.082	2.517	0.012
**Total indirect Effect**	ID→PA→CI	−0.148	0.053	2.806	0.005
	ID→PA→SI	−0.116	0.050	2.307	0.021

Two significant total indirect effects have been identified in the model. If the sign of indirect effect is opposite to that of direct effect, the total effect will be suppressed (Wen and Ye, 2014). The suppressing effect of perceived anonymity accounts for 46.1% of the direct effect between identification and comment intention, and for 31.8% of the direct effect of identification and sharing intention. Perceived anonymity has a significant suppressing effect between identification and two participation factors.

## Discussion and Conclusion

In the current study, we examined user participation in discussions on moral/ethical topics on Chinese SNS. To do so, we constructed a model to describe the influence of identification, perceived anonymity, perceived risk, and information credibility. The measurement model has been confirmed, with acceptable convergent and discriminant validity, path coefficients, and model fit.

### Discussion of Results

Identifiability and perceived anonymity of SNS user identity are not two sides of an organic whole but, rather, two different elements (Chen et al., 2019a). Identifiability reflects the amount of information available on the real identity of the behavioral subject that is identified (Marx, 1999). High identifiability of users leads to low perceived anonymity of identity (Chen et al., 2016, 2019a, b), which is also suggested in the current study with the supportive result for H1a. Furthermore, with the supportive results for H1b, H1c, H2a, and H2b, the current study shows that the influence of online identification and perception of anonymity are both conducive to participation in discussion about moral/ethical violation topics. This result is in accordance with the research on self-disclosure on Weibo (Chen et al., 2016).

When users have control over their identification, perceived anonymity contributes to user participation in discussion on moral/ethical violation topics; when users have control over their perceived anonymity, identification also contributes to participation in discussion of moral/ethical issues. High user identifiability is advantageous in building a sense of identification with the social network identity and enhancing the credibility of the opinions expressed at the same time. The influence of perceived anonymity on speech behavior varies in different application scenarios; there is evidence of a negative influence on the perceived autonomy of sharing behaviors in cyberspace (Yoon and Rolland, 2012), whereas on social media it promotes self-expression without causing a reduction in the perception of self-expression risks (Chen et al., 2016). This leads us to conclude that both identification and perceived anonymity play important roles in participation in discussions of moral/ethical violation topics.

The current study also shows the positive effect of perceived risk on users’ intention to post comments on moral/ethical topics on SNS in China: in the face of an event that raises common ethical concerns, when the level of risk perceived by users is higher, so too is their intention to post comments, as shown by the supportive result for H3a. Reducing cybersecurity risk increasingly depends on information sharing (Goodwin et al., 2015). This result is in line with the statement that risk may add value to SNS in some contexts, as users are motivated to reduce uncertainty (Mitchell, 1999). However, it is not consistent with some research studies which examined the impact of risk on information sharing behavior in other SNS contexts and showed no significant impact on self-disclosure (Chen et al., 2016), and that perceived privacy risk will negatively impact the attitude towards information sharing (Hajli and Lin, 2016). Despite the risks, people participate in relevant social activities to safeguard justice because of their moral sentiments (Gintis et al., 2005). This phenomenon reveals the particularity of participation in moral/ethical-related issues. Participating in discussions on moral/ethical violation topics is out of concern for fairness and justice, as well as to reduce uncertainty. This may be attributed to the neural basis of altruistic punishment in people’s brains (Fehr and Gächter, 2002). Participation in discussions on moral/ethical violation topics on networked social media can be understood as reciprocal behavior with a price, because it is a kind of trial-and-punishment of behavior considered unethical, rather than behavior that is responsive or well-targeted. In the face of unethical events, the behavioral mechanism comes from people’s desire to impose punishment and to gain a sense of satisfaction from participation in imposing punishment (De Quervain et al., 2004). Therefore, perceived risk does not make people shrink from discussion participation, but encourages them to participate in the discussion of moral and ethical issues to some extent. The encouragement from perceived risk does not have a significant effect on sharing intention, but the direction of the impact is consistent with H3b. The relations between perceived risk and intensive participation are of value to explore further.

We also found that information credibility significantly affects participation in discussions on moral/ethics violation topics. This has a positive influence on both posting comments and sharing intention on moral/ethics violation topics, with supportive evidence for H4a and H4b. This result is in accordance with the outcomes from research studies on the impact of information credibility on involvement in discussion and sharing (Zimmer et al., 2010; Ha and Ahn, 2011; Özer et al., 2011). The perception and faith of SNS users regarding the authenticity and reliability of the information source plays a crucial role in the regulation of public speech and spreading of information about moral/ethical violation topics.

### Theoretical Implications

The current study offers some implications that facilitate future research on participating in discussion on moral/ethical violation topics. Public concern and discussion about moral/ethical violation topics is important for regulating negative behaviors and maintaining social justice. User discussions of this kind of behavior on Chinese SNS in recent years plays a dominant role in promoting the advancement of social institutions and governance by drawing the public attention and letting the government know some information. Therefore, investigation into this kind of behavior can lead to a deeper understanding of theories on reputation, altruistic punishment, and regulation of public speech related to social cooperation.

The current study further clarifies the relationship between identifiability and speech behavior. We confirmed the positive impact of identification and perceived anonymity on participation in discussions on moral/ethics-related events on SNS. This lays a foundation for further exploration of the influence of online user identification on behavior. In the current study, unlike previous studies, online participation in moral/ethical violation topics in cyberspace is divided into commenting and information sharing, which are affected differently by the perception of risks in discussions on moral/ethical violation topic. This means that comments on specific events might not necessarily be seen by a user’s social contacts, but information sharing enables user opinions to be seen by a wide range of social contacts. Our research offers a new perspective for viewing the differences between them.

### Practical Implications

The results have practical implications for policy makers, content moderators, and operators of online platforms. Online identification and anonymity perception influence participation in and speech about moral/ethical violation topics, which can have a significant reference value for identity management and internet governance. A network identity policy needs to take into account the reliability of user authentication mechanisms as well as user perception of privacy. This kind of network environment encourages user participation in discussions on sensitive topics. Policy makers should also note that the sense of risk does not necessarily inhibit behavior. In the current study, perceived risk is found to encourage user participation in discussions about moral/ethical violation topics. Successful information efforts require commitment, trust, cooperation, and a clear sense of value (Goodwin et al., 2015). Correct information values are conducive to promoting the sharing and exchange of information.

Besides, the reliability of the topics is very important to the users’ participation in commenting and information sharing. It is necessary to guarantee the reliability of content and information in cyberspace for the network operators and network information providers. It is necessary to regulate the information sources and provide information authentication mechanisms to identify and eliminate false information and rumors and standardize the expression form of information, etc., to promote the discussion and spread of topics.

### Limitations and Future Directions

The current study has the following limitations, which open up some avenues for further research.

First, the research study and survey participants only used Weibo and WeChat in China, neglecting the difference embedded in contexts across China and the West. A trial study aimed to what was being studied, which needs further confirmation. Future research is needed to examine how and to what extent contextual and cultural differences affect the research questions and model. Second, the research provides no empirical support for H3b, which means we failed to confirm a positive impact on users from perceived risk about their sharing intention in discussing moral/ethical issues on Chinese SNS. Although differences between posting comments and sharing information are indicated in our discussion, the reasons and mechanisms should be further explored. Third, although the sample size meets the requirements of PLS SEM research with a degree of representativeness to some extent, the limitations of the study caused by the small and non-representative sample still remain; the size and representativeness of the sample need to be expanded in future research, which will contribute to the generalizability of the findings. In addition, the research sample failed to properly consider differences in age, gender, vocation, economic status, education level, and SNS use; therefore, more variables should be taken into consideration in a future study to enhance the representativeness of the research sample.

People’s participation in discussion on violations of morality and ethics on the internet is taken as a crucial form of public collective activity in the criticism and supervision of society. One of the doubts about the internet real-name system is whether identifiability will impede such power. In the current study, empirical evidence was provided on the positive effects of identification, perceived anonymity, risk perception, and information credibility on users’ participation in discussions on unethical topics in Chinese SNS, with a view to providing a reference point for subsequent academic studies, information management of SNS, and the governance of society.

## Data Availability Statement

The original contributions presented in the study are included in the article/Supplementary Material, further inquiries can be directed to the corresponding author/s.

## Ethics Statement

Ethical review and approval was not required for the study on human participants in accordance with the local legislation and institutional requirements. Written informed consent from the patients/participants or patients/participants legal guardian/next of kin was not required to participate in this study in accordance with the national legislation and the institutional requirements.

## Author Contributions

XC performed the theory analysis and design, and contributed to drafting the manuscript. CH analyzed the data and improved the empirical analysis. YC collected data and improved the conclusions. All authors contributed to the article and approved the submitted version.

## Conflict of Interest

The authors declare that the research was conducted in the absence of any commercial or financial relationships that could be construed as a potential conflict of interest.
